# Biomimetic liposomal nanozymes improve breast cancer chemotherapy with enhanced penetration and alleviated hypoxia

**DOI:** 10.1186/s12951-023-01874-7

**Published:** 2023-04-10

**Authors:** Juanjuan Li, Chunai Gong, Xinlu Chen, Huanhuan Guo, Zongguang Tai, Nan Ding, Shen Gao, Yuan Gao

**Affiliations:** 1 School of Pharmacy & Zhong Shan Hospital, Fudan University, Shanghai, 201206 China; 2 Department of Pharmacy, Changhai Hospital, Second Military Medical University, Shanghai, 200433 China; 3 Department of Pharmacy, Shanghai Ninth People’s Hospital, School of Medicine, Shanghai Jiao Tong University, Shanghai, 200011 P. R. China; 4 Shanghai Skin Disease Hospital, Tongji University School of Medicine, Shanghai, 200443 China

**Keywords:** Breast cancer, Chemotherapy, Biomimetic nanoparticles, Hypoxia, Penetration

## Abstract

**Background:**

Doxorubicin (Dox) has been recommended in clinical guidelines for the standard-of-care treatment of breast cancer. However, Dox therapy faces challenges such as hypoxia, acidosis, H_2_O_2_-rich conditions and condensed extracellular matrix in TME as well as low targeted ability.

**Methods:**

We developed a nanosystem H-MnO_2_-Dox-Col NPs based on mesoporous manganese dioxide (H-MnO_2_) in which Dox was loaded in the core and collagenase (Col) was wrapped in the surface. Further the H-MnO_2_-Dox-Col NPs were covered by a fusion membrane (MP) of inflammation-targeted RAW264.7 cell membrane and pH-sensitive liposomes to form biomimetic MP@H-MnO_2_-Dox-Col for *in vitro* and *in vivo* study.

**Results:**

Our results shows that MP@H-MnO_2_-Dox-Col can increase the Dox effect with low cardiotoxicity based on multi-functions of effective penetration in tumor tissue, alleviating hypoxia in TME, pH sensitive drug release as well as targeted delivery of Dox.

**Conclusions:**

This multifunctional biomimetic nanodelivery system exhibited antitumor efficacy *in vivo* and *in vitro*, thus having potential for the treatment of breast cancer.

**Supplementary Information:**

The online version contains supplementary material available at 10.1186/s12951-023-01874-7.

## Background

Breast cancer is the leading cancer in women worldwide, accounting for approximately about 31% of all new cancer cases in women in the United States in 2023 [1]. Hypoxia is a prominent characteristic of breast cancer [2], which is caused by the imbalance between insufficient oxygen supply results from the chaotic vascular structure and increased oxygen consumption results from the vigorous metabolism of tumor cells [3–5]. The hypoxic promotes tumor malignancy and reduces sensitivity to chemotherapy, radiotherapy, as well as photodynamic therapy [6–8]. Moreover, hypoxia also leads to chronic over activation of hypoxia-inducible-factor-1 (HIF-1), activating glucose transporters and glycolytic enzymes with increased levels of lactic acid and acidosis, and inducing H_2_O_2_ production [9, 10]. These characteristics together promote tumor metastasis, resistance to therapies, leading to treatment failure.

The clinical treatment of breast cancer usually includes various combinations of surgery, radiotherapy, targeted therapy, chemotherapy, and endocrine therapy, etc. [11]. As a first-line chemotherapy drug, doxorubicin (Dox) is commonly used in the treatment against breast cancer. However its use is limited by drug resistance and side effects. Elleviating hypoxia could be a potential strategy to solve the challenge. Many therapeutic strategies for breast cancer hypoxia were reported including HIF inhibitors [12], hypoxia-activable prodrug [13] and in situ oxygenating to modulate TME [14]. Usually, there two main approaches for normalizing tumor oxygen supply. One strategy is direct delivery oxygen into the tumor using artificial oxygen carriers with limited O_2_ loading capacity, such as perfluorocarbons and hemoglobin [15, 16]. The other strategy is to indirectly producing oxygen in situ by enzymes such as catalase or the manganese dioxide nanozyme [17–19]. Manganese dioxide (MnO_2_) nanoparticles (NPs) were reported to have a high specificity and reactivity toward H_2_O_2_, generating O_2_ and H_2_O under acidic TME conditions attenuating hypoxia and regulation of pH [20–22]. Moreover, Mn^2+^ ions, products of MnO_2_ and important trace elements, can be efficiently metabolized *in vivo* and are used as magnetic resonance imaging contrast agents, aiding the diagnosis and treatment of cancer [23, 24]. MnO_2_ NPs have been demonstrated to be safety without long-term toxicity in cancer therapy *in vivo* [25, 26]. In particular, hollow MnO_2_ (H-MnO_2_) nanostructures have a large specific surface area and high pore volume, providing excellent drug loading and delivery properties [27, 28].

Another challenge for Dox therapy is the condensed extracellular matrix (ECM) with high collagen content dramatically hindering the diffusion of nanocarriers to deeper tumor sites [29–31]. Various studies have shown that collagenase (Col) can be modified onto the surface of nanocarriers, promoting their diffusion [32–35]. However, as enzymes are labile entities that can be inactivated in the bloodstream, finding carriers suitable for enzyme delivery remains a challenge [36, 37]. Additionally, the absorption of plasma proteins to the surface of nanomedicines administered intravenously accelerates recognition and phagocytosis of the reticuloendothelial system, resulting in most nanomedicines rarely reaching the tumor site [36, 38]. The use of biomimetic strategies can solve the problem, with intrinsic biocompatibility, extended blood retention, high biodegradability, and precise tumor tissue targeting [39–41]. Previous studies have shown that macrophages can bind to cancer cells through specific ligand interactions (α4 integrins and vascular cell adhesion molecule-1 (VCAM-1), and NPs camouflaged with macrophage membranes can enhance the targeting of NPs in lung metastasis of breast cancer treatment [42, 43], owing to the inflammatory tendency of macrophages and the retention of membrane proteins [42, 44].

**Scheme 1:**
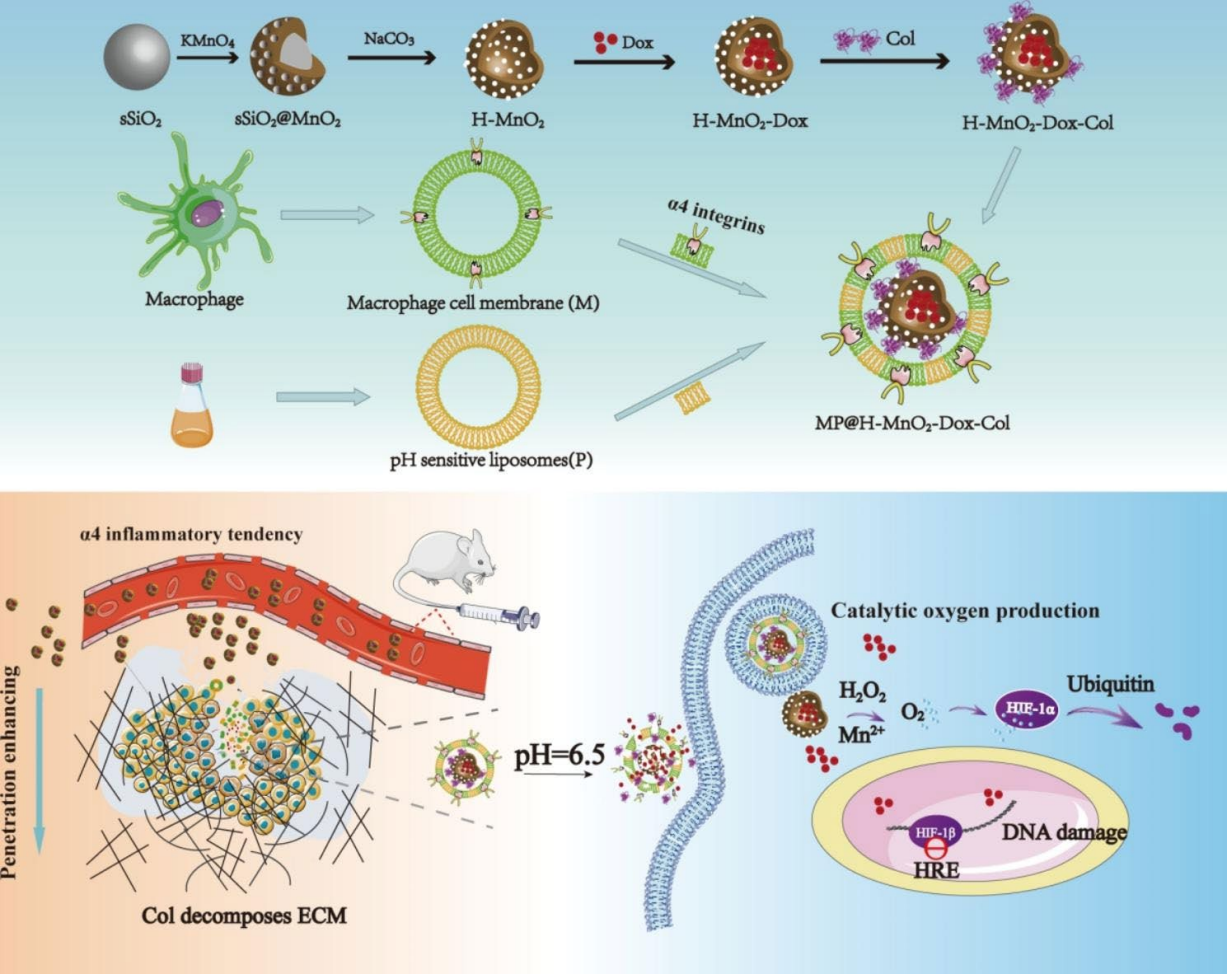
Schematic illustration of the mechanism of MP@H-MnO_2_-Dox-Col NPs with enhanced *in vivo* chemotherapy

In this study, a nanosystem H-MnO_2_-Dox-Col NPs was developed based on H-MnO_2_ in which Dox was loaded in the core and collagenase (Col) was wrapped in the surface. Further the H-MnO_2_-Dox-Col NPs were covered via a fusion membrane (MP) of inflammation-targeted RAW264.7 cell membrane (M) and pH-sensitive liposomes (P) to form biomimetic MP@H-MnO_2_-Dox-Col. We hypotheses that the MP@H-MnO_2_-Dox-Col NPs could be targeted delivery to tumor due to the MP’s cancer-homing and inflammation targeting ability. ECM degradation at the tumor site might be confirmed with the help of Col, and thus endowing the nanosystem with acidic TME sensitivity, promoting the penetration of NPs. Moreover, H-MnO_2_ could release Dox at an acidic TME, alleviate hypoxia and regulation of pH, and therefore enhance the effect of Dox. For this purpose, MP@H-MnO_2_-Dox-Col NPs were prepared (Scheme 1), and their enhanced effects and possible mechanisms for breast cancer therapy were further evaluated *in vitro* and *in vivo*.

## Results

### Characterization of MP@H-MnO_2_-Dox-Col NPs

**Fig. 1:**
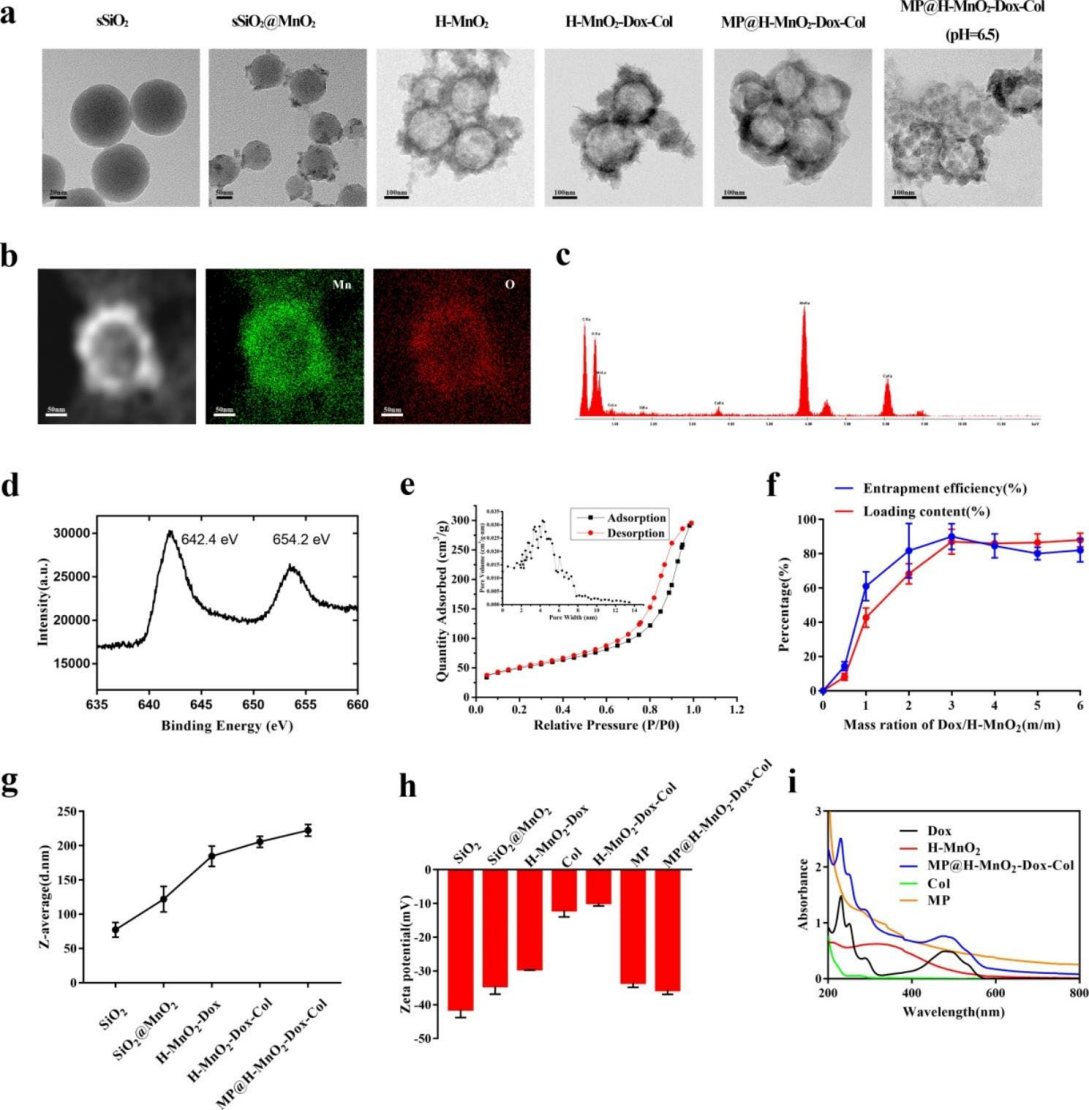
Synthesis and characterization of MP@H-MnO_2_-Dox-Col nanoparticles (NPs). (a) Transmission electron microscopy images (TEM) of the NPs at each step of preparation and MP@H-MnO_2_-Dox-Col NPs after treatment in pH 6.5 buffers for 2 h. (b) High-angle annular dark-field scanning TEM (HAADF-STEM) images and elemental mapping for H-MnO_2_. (c) Energy dispersive X-ray spectroscopy data of H-MnO_2_. (d) X-ray photoelectron spectroscopy spectrum of H-MnO_2_. (e) Pore-size distribution curve (inset) and N_2_ adsorption/desorption isotherms of the H-MnO_2_ sample. (f) Dox loading rate and encapsulation rate in H-MnO_2_ at different feeding Dox: H-MnO_2_ ratios. Data are presented as the mean ± standard deviation (SD) (n = 3). (g) Particle size and (h) the surface charge potential of different NPs during the preparation process. (i) UV/VIS/NIR spectrum of the aqueous dispersion of different NPs

The transition from solid silica to mesoporous MnO2 was observed using Transmission Electronic Microscopy (TEM). TEM images of H-MnO_2_, H-MnO_2_-Dox-Col and MP@H-MnO_2_-Dox-Col NPs revealed the spherical morphology. Moreover, MP@H-MnO_2_-Dox-Col NPs retained the hollow structure of H-MnO_2_ with a layer of cell membrane outside, confirming the successful membrane coated (Fig. 1a). The average particles size of MP@H-MnO_2_-Dox-Col NPs was ~ 220 nm. Moreover, the hollow nanostructure was also confirmed using high-angle annular dark-field scanning TEM (HAADF-STEM)-based elemental mapping (Fig. 1b) and STEM-energy dispersive X-ray spectroscopy elemental mapping analysis (Fig. 1c), with a high distribution of Mn and O in the analyzed area. Treatment of MP@H-MnO_2_-Dox-Col NPs with an acidic solution for 2 h resulted in the disintegration of particles (Fig. 1a), which may be due to the acid instability of H-MnO_2_ and the acid sensitivity of the hybrid membrane. Two characteristic peaks of Mn 2p at 652.6 eV (Mn (IV) 2p1/2) and 641 eV (Mn (IV) 2p3/2) were observed in the spectrum generated using X-ray photoelectron spectroscopy (Fig. 1d) and were assigned to spin–orbit peaks of MnO_2_ [45], revealing the + 4 valence state of manganese in the NPs and the successful formation of MnO_2_. Brunauer–Emmett–Teller (BET) analysis showed that the surface area and average pore diameter of H-MnO_2_ were 175.696 m^2^/g and 4.5 nm, respectively (Fig. 1e). The mesoporous structure of MnO_2_ NPs has been reported as appropriate for the efficient loading of small-molecule drugs [46].

After the characterization of NPs, Dox was loaded into H-MnO_2_ at different concentrations (Fig. 1f). At a mass ratio (Dox: H-MnO_2_) of 3:1, the encapsulation efficiency and drug loading reached relatively stable values of up to 90% and 87%, respectively. This feeding ratio was selected for subsequent experiments. Hydrodynamic diameter and zeta potential results are shown in Fig. 1g and h. The average particles size of the MP@H-MnO_2_-Dox-Col NPs was approximately 220 nm, which is in good agreement with that of TEM results. The observed diameter increase (10–20 nm) between MP@H-MnO_2_-Dox-Col NPs and H-MnO_2_-Dox-Col NPs could be due to the distinct thickness of the phospholipid bilayer membrane [47, 48]. The zeta potential of MP@H-MnO_2_-Dox-Col NPs was similar to that of the hybrid MP membrane materials. These results further confirmed the successful coating of the hybrid membrane. As can be observed from the absorption spectra (Fig. 1i), the characteristic absorption peak at 480 nm in the spectrum of MP@H-MnO_2_-Dox-Col NPs matched well with that of Dox. The new absorption band around 300–400 nm could be attributed to the surface plasmon band of MnO_2_ nanoclusters [49], further confirming the successful synthesis of MP@H-MnO_2_-Dox-Col NPs.

### Physicochemical characterization of fusogenic MP

**Fig. 2:**
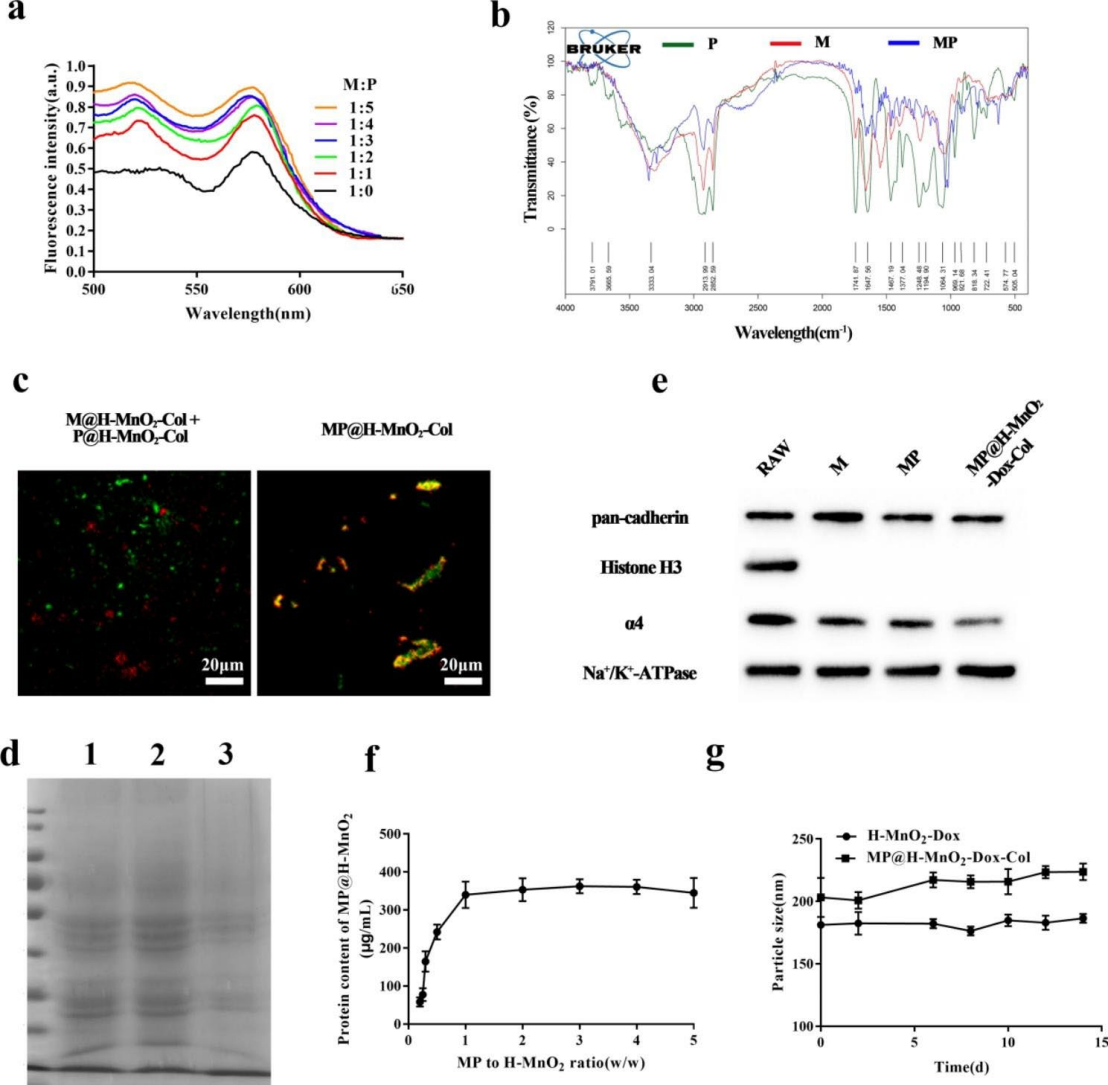
Characterization of hybrid membrane MP. (a) The RAW264.7 cell membranes labeled with DOPE-RhB/C6-NBD were fused with increasing amounts of liposomes, and their fluorescence spectra were recorded. M:P indicates the weight ratio of RAW264.7 cell membrane proteins to liposomes. (b) The Fourier transform infrared (FTIR) spectra of M, P, and MP confirmed the retention of RAW264.7 cell membrane proteins in MP. (c) Representative laser-scanning microscopy images of the M@H-MnO_2_-Col NPs and P@H-MnO_2_-Col NPs mixture and fused MP@H-MnO_2_-Col NPs (scale bars = 40 μm). (d) Profiles of proteins in M (1), MP (2), and MP@H-MnO_2_-Dox-Col NPs (3) determined via SDS-PAGE. (e) Western blot analysis of RAW264.7 cells, M, MP, and MP@H-MnO_2_-Dox-Col NPs for characteristic RAW264.7 membrane marker α4 (Na^+^-K^+^-ATPase was used as a reference protein). (f) Optimization of MP-to-H-MnO_2_ weight ratios (w/w) via BCA analysis. (g) Z-average size of H-MnO_2_-Dox NPs and MP@H-MnO_2_-Dox-Col NPs over 15 days in water

To verify the fusion properties, M was doped with a pair of FRET dyes, and increasing amounts of P were added. As shown in Fig. 2a, fluorescent signal recovery was observed at around 534 nm owing to the interaction of the two materials weakening the FRET. An M: P weight ratio of 1:1 was used for subsequent experiments. In addition, we identified the vibrational modes and chemical signatures of M, P, and MP using FT-IR spectroscopy (Fig. 2b). Specifically, the band at 1,700–1,600 cm^− 1^ corresponded to C = O stretching vibrations and that at 1,600–1,500 cm^− 1^ was ascribed to NH bending with C-N stretching vibrations. Similar typical protein absorption bands were present in the groups of M relative to those found in the MP group, demonstrating the incorporation of protein components in the hybrid membrane. Furthermore, M and P were stained with DiR and DiO fluorescent dyes, respectively, and fluorescence was observed using a CLSM (Fig. 2c). The overlay of fluorescence on MP@H-MnO_2_-Col NPs and the distinct fluorescent puncta on M@H-MnO_2_-Col NPs + P@H-MnO_2_-Col NPs confirmed the fusion of M and P.

### Membrane protein characterization

Our previous studies have also demonstrated the targeting ability of macrophage cell membranes [39, 43]. The protein profiles of MP@H-MnO_2_-Dox-Col NPs were analyzed using SDS-PAGE (Fig. 2d). Compared with M (1), MP (2) and MP@H-MnO_2_-Dox-Col NPs (3) retained RAW264.7 cells membrane proteins well. Specific protein markers in different samples were detected using western blotting (Fig. 2e). α4 integrins expressed on RAW264.7 cells are important for macrophage adhesion and activation. Highly expressed α4 integrins were detected on RAW264.7 cells, M, MP, and MP@H-MnO_2_-Dox-Col NPs. Moreover, the cellular membrane marker pan-cadherin was also found on RAW264.7 cells, M, MP, and MP@H-MnO_2_-Dox-Col NPs, while the nuclear marker histone H3 was absent in MP@H-MnO_2_-Dox-Col NPs. These results indicated successful preparation of the hybrid membrane and the successful coating of the NPs surface. Furthermore, the BCA assay results presented in Fig. 2f indicated that the optimal hybrid membrane-to-H-MnO_2_ ratio was 1:1. Finally, we explored the stability of MP@H-MnO_2_-Dox-Col NPs and H-MnO_2_-Dox NPs dissolved in PBS solution using DLS. As shown in Fig. 2g, we found that both of MP@H-MnO_2_-Dox-Col NPs and H-MnO_2_-Dox NPs maintained a stable size for the 2-week duration of the study.

### ***In vitro*** H^+^- and H_2_O_2_-triggered dox release

**Fig. 3:**
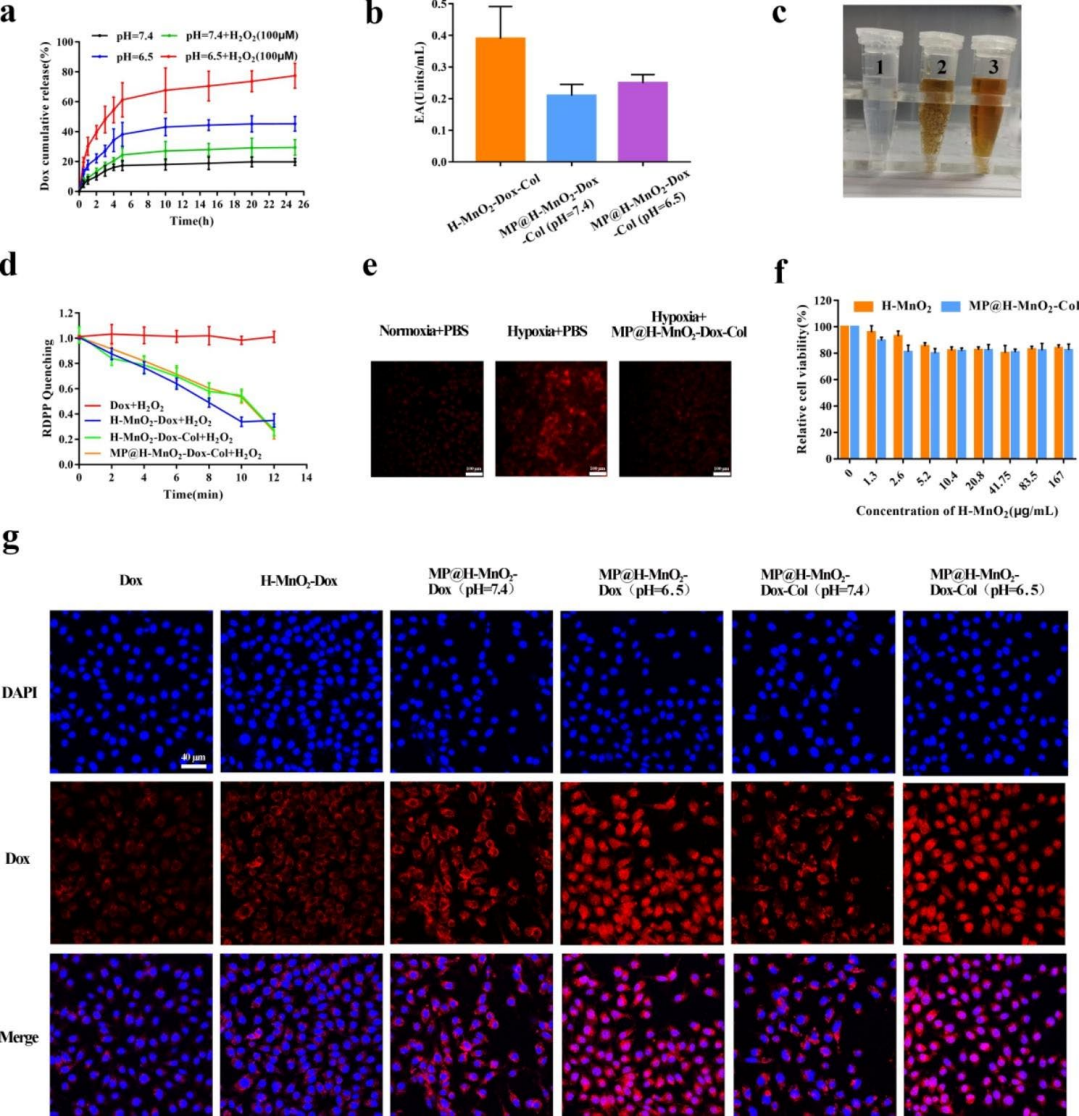
Characterization of functional properties *in vitro*. (a) Dox release profiles from MP@H-MnO_2_-Dox-Col NPs with or without 100 µM H_2_O_2_ at different pH values. Data are presented as the mean ± SD (n = 3). (b) Relative viabilities of 4T1 cells after incubation with various concentrations of H-MnO_2_ in the dark for 24 h. Data are presented as the mean ± SD (n = 6). (c) Quantification of the enzyme activity (EA) of collagenase-modified NPs (n = 3). (d) Digital images of MP@H-MnO_2_-Dox-Col NPs with or without H_2_O_2_ to measure O_2_ generation: (1) only H_2_O_2_; (2) H_2_O_2_; (3) without H_2_O_2_; (e) The generation of oxygen as determined based on quenched RDPP fluorescence; (f) Fluorescence images of MP@H-MnO_2_-Dox-Col NPs induced hypoxia attenuation; (g) CLSM images of intracellular distribution of Dox in each group (scale bars = 40 μm)

The exploration of the bioresponsiveness of nanomaterials is important [50]. MnO_2_ has been proven to decompose under H^+^ and H_2_O_2_ conditions, and, according to previous reports, H_2_O_2_ is found at concentrations of 10–100 µM in most solid tumors [51]. Further, H^+^ microenvironment is characteristic of the TME [52]. Hence, the release behavior of Dox from MP@H-MnO_2_-Dox-Col NPs was recorded at pH 7.4 or 6.5, with or without the addition of H_2_O_2_ (100 µM). As shown in Fig. 3a, less than 20% of the Dox molecules within MP@H-MnO_2_-Dox-Col NPs was released at pH = 7.4, and approximately 30% was released at pH = 7.4 with 100 µM H_2_O_2_. Sustained Dox was observed at pH 6.5 over 12 h, with an increase of up to 45.23%. Moreover, in the presence of H_2_O_2_ and pH 6.5, the amount of Dox released from MP@H-MnO_2_-Dox-Col NPs gradually reached > 70% (*p* < 0.01) within 12 h, which was considerably higher than that observed for the control. Taken together, characteristics of H^+^ and H_2_O_2_ at the tumor site were beneficial for the “on-demand” release of our NPs.

### Enzymatic activity study

As the collagen-rich ECM can be degraded by Col for enhanced drug delivery, we assessed the collagenase activity of NPs using the EnzChekt™ Gelatinase/Collagenase Assay Kit (E12055). For this assay, fluorescently-labeled collagen was used as the substrate, and Col activity was detected based on the increase in fluorescence. As shown in Fig. 3b, after pretreatment of MP@H-MnO_2_-Dox-Col NPs at pH 6.5 for 30 min, the enzymatic activity was 0.25 U mL^− 1^, which was approximately 1.2 times higher than that in the pH 7.4 group. Meanwhile, the enzyme efficiency in the membrane-coated group at pH 7.4 was reduced by nearly 45% compared with that of the H-MnO_2_-Dox-Col NPs group. The above results indicate that the hybrid membrane plays a crucial role in preventing enzyme inactivation during transportation as well as in the effective delivery of Col into the tumor site.

### ***In vitro*** detection of O_2_ generation

As MnO_2_ has catalase-like activity, catalyzing the conversion of H_2_O_2_ to O_2_, we treated the MP@H-MnO_2_-Dox-Col NPs with H_2_O_2_ under acidic conditions to evaluate O_2_ generation. Results presented in Fig. 3c indicate the immediate generation of numerous bubbles. To quantify the production of oxygen, different samples were detected using an RDPP probe, and the fluorescence was recorded (Fig. 3d). While the MnO_2_-containing NPs showed rapid fluorescence quenching, the free Dox group maintained constant fluorescence intensity, indicating that the production of O_2_ was attributed to MnO_2_-mediated catalysis. Furthermore, to evaluate the hypoxia attenuation, we assessed the fluorescence of the NPs *in vitro* by imaging 4T1 cells under a fluorescence microscope. The normoxic group, in which fluorescence density was low, was used as a control. The anoxic group exhibited a strong fluorescence signal over a large area, whereas the MP@H-MnO_2_-Dox-Col NPs treatment group showed a fluorescence signal similar to that in the normoxic group (Fig. 3e). The above evidence suggests that our NPs can significantly alleviate hypoxia within the tumor site.

### The biocompatibility and cellular distribution of MP@H-MnO_2_-dox-col NPs

The biocompatibility of blank MP@H-MnO_2_-Col NPs was investigated using a cell viability assay. 4T1 cells exhibited no obvious cytotoxicity after treatment with blank NPs at concentrations ranging from 0 to 167 µg/mL (Fig. 3f). In addition, the cellular distribution of different formulations was analyzed using a CLSM. The fluorescence signal in the MP-coated group was stronger than that in the free Dox and bare H-MnO_2_-Dox NPs (Fig. 3g), which may be explained by the specificity of the interaction between RAW264.7 cell membranes and tumor cell membranes [43]. We further observed that the distribution of NPs at pH 6.5 was greater than that at pH 7.4, confirming the acid-sensitive nature of our nanosystem. However, the distribution was not significantly different between the MP@H-MnO_2_-Dox NPs and the MP@H-MnO_2_-Dox-Col NPs groups at both pH 6.5 and 7.4, which may be due to the lack of abundant collagen in 2D cellular cultures.

### Penetration in 3D tumor spheroids

**Fig. 4:**
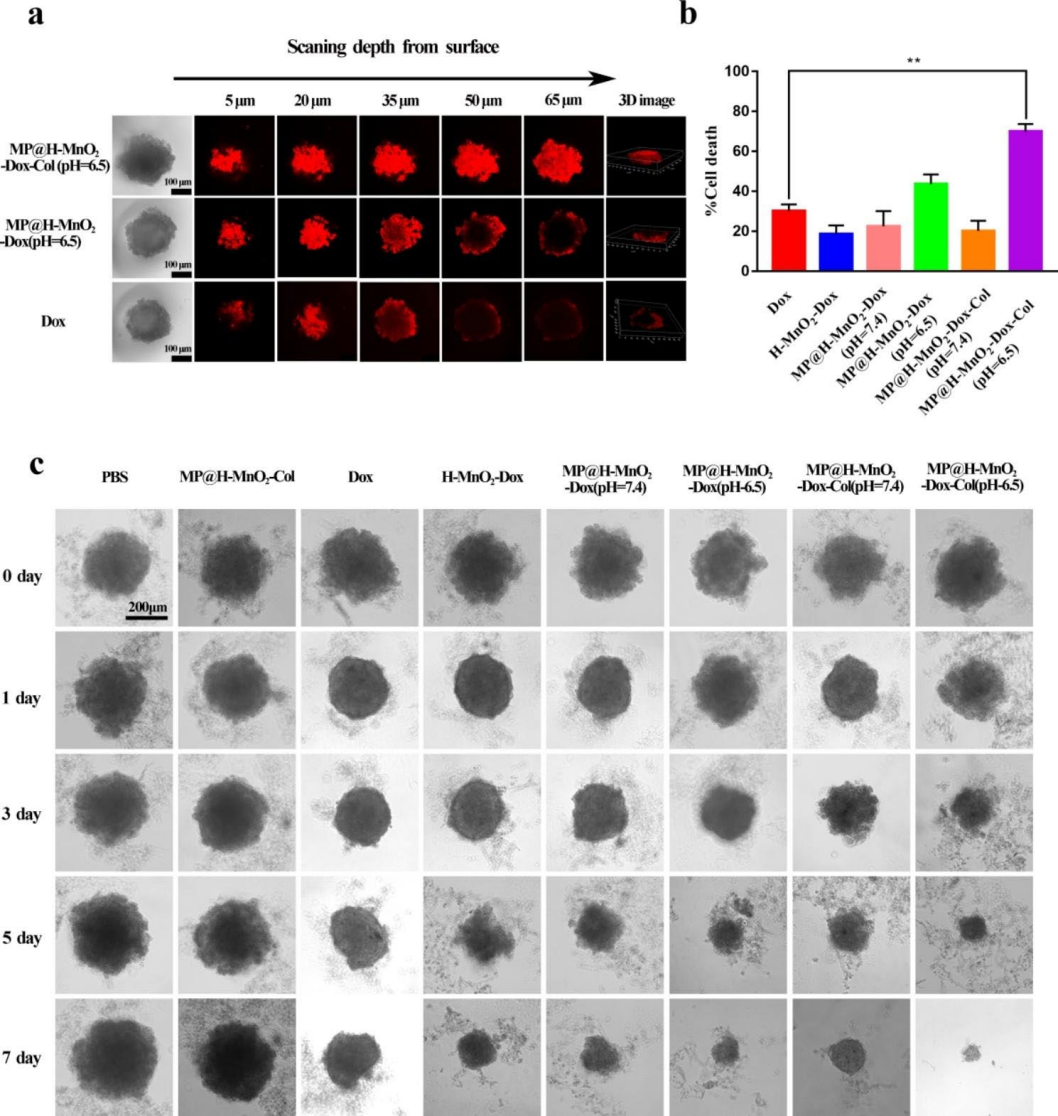
*In vitro* efficacy of NPs in the 3D tumor spheroid model. (a) CLSM images showing *in vitro* penetration of MP@H-MnO_2_-Dox-Col NPs (pH = 6.5), MP@H-MnO_2_-Dox NPs (pH = 6.5) and free Dox in 3D-cultured 4T1 multicellular spheroids. (b) Spheroid cytotoxicity under treatment with the different NPs formulations for 72 h was evaluated *via* LDH assays. (c) Representative images of 4T1 3D tumor spheroids incubated with different NPs treatment on different days

To evaluate the ECM-degrading ability of Col and the penetration of NPs, we simulated the cell matrix and the interaction between cells using tumor spheroids. Faint fluorescence was only observed at the edges of the spheroids in the group treated with free Dox (Fig. 4a). Meanwhile, the tumor spheroids incubated with MP@H-MnO_2_-Dox NPs (pH = 6.5) showed comparatively strong red fluorescence in the outer ring (50 μm depth) but not in deeper areas, indicating that the drug could not enter deep into the tumor to exert a therapeutic effect. Conversely, permeability of MP@H-MnO_2_-Dox-Col NPs (pH = 6.5) was comparatively strong, as the red fluorescence was almost completely distributed inside the tumor sphere at a scanning depth of 65 μm. The intensity and distribution of the red fluorescence were also observed clearly in the 3D image.

### LDH cytotoxicity assay

To further verify the cytotoxicity and intracellular delivery of MP@H-MnO_2_-Dox-Col NPs, we performed several analyses on 3D tumor multicellular spheroids (MCSs), which are an ideal *in vitro* model to mimic the real tumor microenvironment and to test the developed pH-sensitive, permeation-enhancing drug delivery system [53, 54]. Follow-up experiments were conducted when the diameter of the MCSs reached approximately 250 μm. An LDH assay showed that free Dox induced 30.2 ± 3.3% of cell death, whereas the cell death was slightly reduced in the other treatment conditions: 18.6 ± 4.3% for H-MnO_2_-Dox NPs; 22.5 ± 7.6% for MP@H-MnO_2_-Dox NPs at pH = 7.4; and 20.2 ± 5.0% for MP@H-MnO_2_-Dox-Col NPs at pH = 7.4 (Fig. 4b). These results may be attributed to the small hindrance of free Dox and the low penetration of other NPs in the tumor spheroids, which is consistent with previous studies [55, 56]. The MP@H-MnO_2_-Dox-Col NPs (pH = 6.5; 69.9 ± 3.7%) showed good efficacy, which may be attributed to the acid-sensitive cleavage of the hybrid membraneas well as exposed collagenase to degrade the matrix and in turn would promote the penetration of NP and the greater efficacy.

### Inhibition assay of 3D tumor spheroids

The effect of the synthesized NPs was further investigated by observing the morphological characteristics of the tumor spheroids under a microscope. As shown in Fig. 4c, the MP@H-MnO_2_-Col NPs group showed no significant inhibitory effect on the growth of the tumor spheroid, indicating that the empty carrier did not exert any adverse effects on 4T1 cells, which was consistent with the previous 2D assay results. During the first 3 days, the growth of tumor spheroids was restrained by treatment with free Dox; however, increased tumor volume was observed after that. In contrast to the Dox group, the H-MnO_2_-Dox NPs, MP@H-MnO_2_-Dox NPs (pH = 7.4 or 6.5), and MP@H-MnO_2_-Dox-Col NP (pH = 7.4) groups exhibited tumor growth inhibition after several days of treatment, demonstrating that the enhanced targeting ability of the hybrid membrane promotes the uptake of more Dox into cells. It should be noted that the volume of the tumor spheroids in the MP@H-MnO_2_-Dox-Col NPs (pH = 6.5) group decreased from day 1 to day 7, implying that exposure to collagenase under acidic conditions promotes the penetration of NPs into deep tumors, which is accompanied by strong cellular uptake by the hybrid membrane. These results were consistent with the results of tumor spheroid permeation findings, suggesting that the degradation of ECM by collagenase promotes NPs penetration and increases cellular uptake of NPs, thus enhancing the benefits of chemotherapy.

### ***In vitro*** distribution and antitumor therapy study

**Fig. 5:**
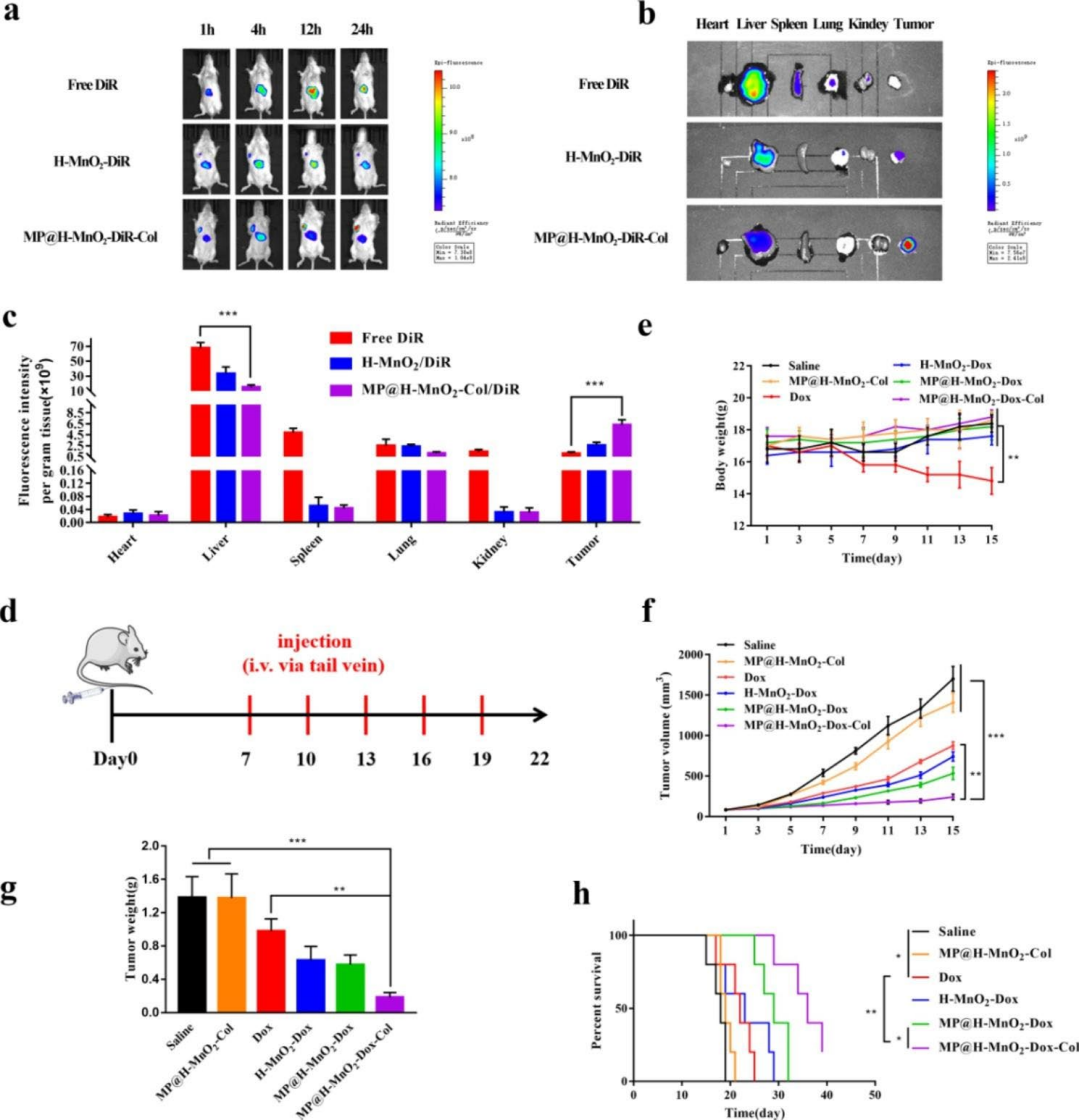
Biodistribution and antitumor effect *in vivo.* (a) Digital photo of breast cancer model. (b) *In vitro* biodistribution in 4T1 tumor-bearing mice after intravenous injection of free DiR, H-MnO_2_-DiR, and MP@H-MnO_2_-Col-DiR was observed over various time intervals. (c) *Ex vivo* fluorescence images of tumor and organs collected from each group were taken at 24 h post-injection. (d) Fluorescence quantitative analysis of DiR distribution in the *ex vivo* tumor and organ. (e) *In vivo* administration protocol for different NPs treatment. (f) Mean body weights of mice from each treatment group. (g) Tumor growth curves of mice after treatments. (h) Weights of tumors excised after 15 days of treatment. (i) Survival of mice from different treatment groups. (*p*-values were calculated via the Student’s *t* test: *p < 0.05, **p < 0.01, ***p < 0.001, n = 5)

*In vitro* drug distribution studies are necessary to evaluate the safety and targeting of NPs. *In vivo* drug distribution was observed using an IVIS imaging system. Tumor model was successfully constructed in BALB/c mice (Fig. 5a). Fluorescence in the free DiR group was mainly concentrated in the liver (Fig. 5b). The fluorescence in the H-MnO_2_-DiR and MP@H-MnO_2_-DiR-Col groups was also localized at the tumor site and increased over time, and the fluorescence of MP@H-MnO_2_-DiR-Col in the tumor was stronger and lasted longer than that of H-MnO_2_-DiR, indicative of the prominent targeting ability of MP@H-MnO_2_-DiR-Col. Furthermore, the tumor and main organs were extracted for fluorescence imaging (Fig. 5c). The free DiR group presented higher fluorescence signals in the liver and spleen, which was in line with the results of previous studies [57]. Stronger fluorescence at the tumor site was observed in the MP@H-MnO_2_-DiR-Col group compared to that in the H-MnO_2_-DiR group, while no DiR fluorescence was observed at the tumor site in the free DiR group. A quantitative analysis showed consistent trends with the distribution *in vivo* of DiR (Fig. 5d). Altogether, these results demonstrate that the hybrid membrane retained the advantages of tumor homing and RES [44] evasion of M, while also endowing NPs with targeting capability, thus increasing NPs concentration at the tumor sites.

To evaluate *in vivo* the therapeutic effects and biosafety of NPs, changes in the tumor volume and body weight of mice were recorded according to the defined schedule (Fig. 5e). As shown in Fig. 5f, body weight increased steadily in the saline, blank MP@H-MnO_2_-Col NPs, H-MnO_2_-Dox NPs, MP@H-MnO_2_-Dox NPs, and MP@H-MnO_2_-Dox-Col NPs groups. However, body weight of Dox group decreased over time, possibly owing to the heart toxicity of Dox. This indicated that H-MnO_2_-Dox NPs and hybrid membrane-coated NPs exerted no significant systemic toxicity. In addition, tumor size rapidly increased in the saline and blank MP@H-MnO_2_-Col NPs groups, while free Dox inhibited tumor growth to some extent. The volume of tumors in MP@H-MnO_2_-Dox-Col NPs-treated mice was remarkably smaller than that in other groups (Fig. 5g). This tendency was also observed in the tumor weight changes graph (Fig. 5h), potentially owing to the efficient delivery of the carrier and the MnO_2_-mediated alleviation of hypoxia. In addition, the survival time results (Fig. 5i) showed that MP@H-MnO_2_-Dox-Col NPs presenting remarkably longer survival period of 36 days, which was higher than that of the saline group (18 days), Dox group (22 days), and MP@H-MnO_2_-Dox group (32 days), suggesting that MP@H-MnO_2_-Dox-Col NPs potently inhibited tumor growth.

### ***In vivo*** assessment of NPs therapeutic effects, collagenase activity, and hypoxia alleviation

**Fig. 6:**
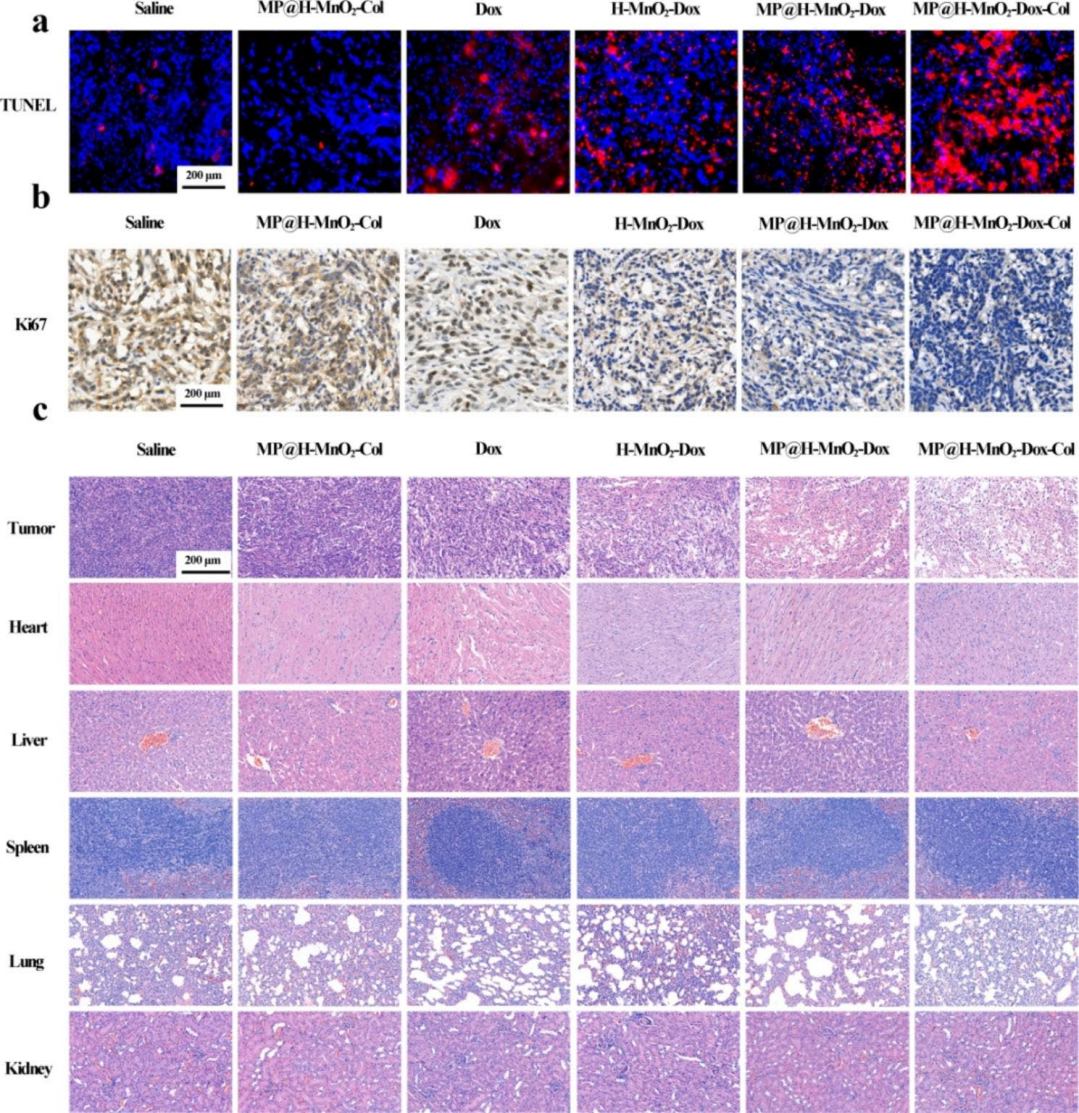
*In vivo* antitumor efficacy and safety evaluation. (a) Immunofluorescence images of TUNEL-stained tumor slices. (b) Ki67 staining of tumor tissues. (c) H&E staining of the tumor, heart, liver, spleen, lung, and kidney

To further investigate the antitumor effects of NPs, an immunofluorescence assay was performed using TUNEL staining, which showed large areas of apoptosis in tumor tissue in the MP@H-MnO_2_-Dox-Col group (Fig. 6a). Similarly, immunohistochemical staining for Ki67 (Fig. 6b) revealed the lowest level of proliferating Ki67-positive tumor cells (depicted in brown) in the MP@H-MnO_2_-Dox-Col group. In addition, H&E staining of the tumor revealed that the group treated with MP@H-MnO_2_-Dox-Col NPs exhibited the greatest tumor cell damage and apoptosis. Simultaneously, H&E staining revealed no noticeable histological alterations in any of the major organs (heart, liver, spleen, lung, and kidney) in the MP@H-MnO_2_-Dox-Col NPs-treated group, in contrast to the myocardial injury observed in free Dox group, confirming that the MP@H-MnO_2_-Dox-Col NPs exhibited lower Dox-associated systemic toxicity.

**Fig. 7:**
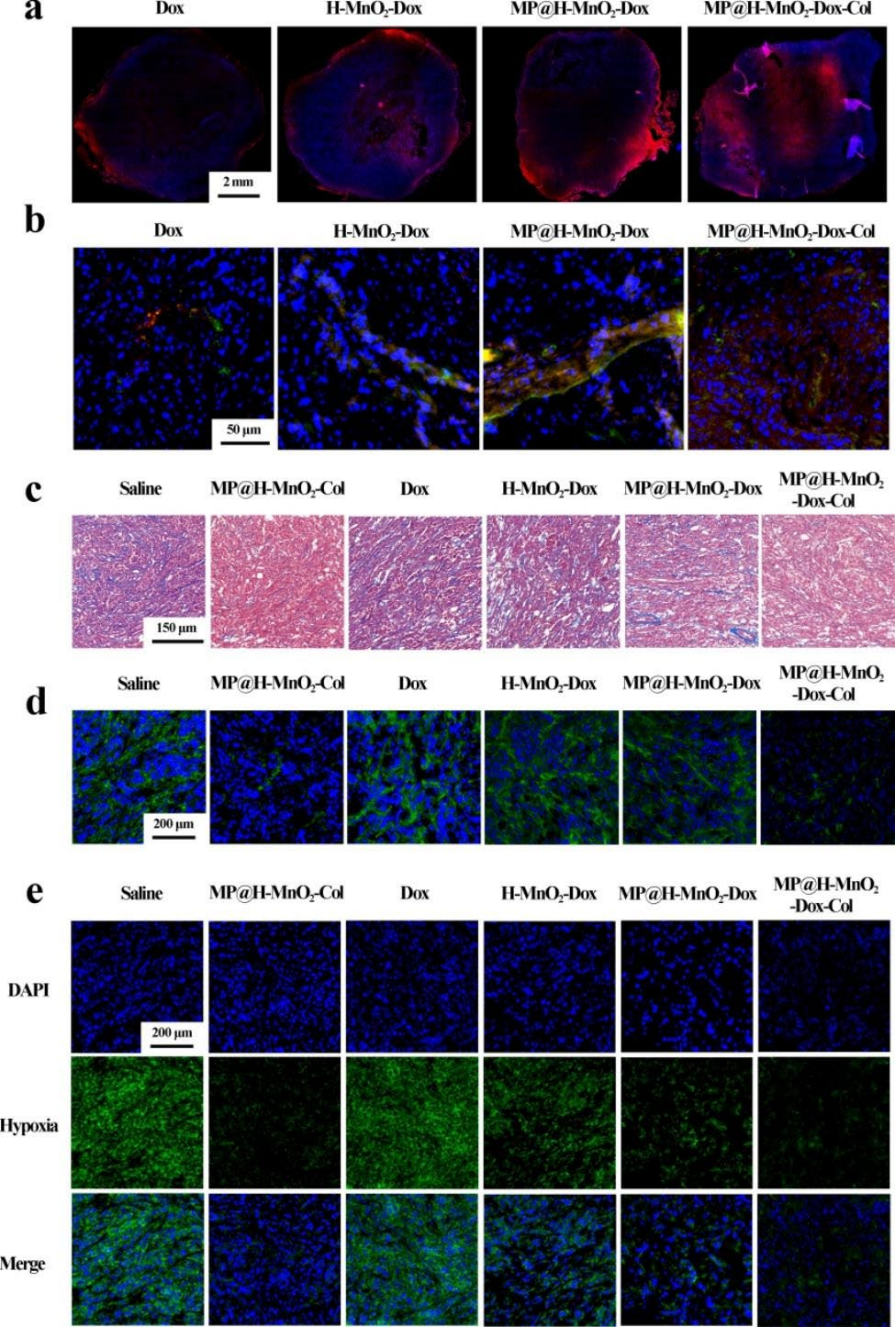
Study on promoting penetration and alleviating hypoxia of NPs. (a) Scan images of tumor tissue, Dox (red). (b) Blood vessels are indicated by CD31 staining (green). (c) Masson’s trichrome analysis of tumors, showing collagen fibers (blue), muscle fibers, cellulose, and red blood cells (red). (d) Representative immunofluorescence images of collagen I (green). (e) Immunofluorescence images of hypoxic areas within tumors. The nuclei and hypoxic areas were stained with DAPI (blue) and an anti-pimonidazole antibody (green), respectively

Given the previous *in vitro* results showed that the NPs exhibit collagenase activity, we further studied the effect of collagenase *in vivo*. For this purpose, tumors with treatment of Dox, H-MnO_2_-Dox NPs, MP@H-MnO_2_-Dox NPs, and MP@H-MnO_2_-Dox-Col NPs were sectioned and scanned for imaging. As shown in Fig. 7a, only red fluorescence was observed at the outer edge of tumors in mice treated with Dox, H-MnO_2_-Dox NPs, and MP@H-MnO_2_-Dox NPs, whereas red fluorescence was also observed in the interior of tumors in mice treated with MP@H-MnO_2_-Dox-Col NPs. Moreover, we studied the distribution of NPs around tumor vessels (Fig. 7b). Dox fluorescence was relatively low in the blood vessels of the Dox group, and increased in the groups treated with H-MnO_2_-Dox NPs and MP@H-MnO_2_-Dox NPs. The fluorescence increase was more pronounced in the MP@H-MnO_2_-Dox-Col NPs group, meanwhile, red Dox fluorescence was also widely distributed in tumor. These results are attributed to the *in vivo* activity of collagenase and the specific tumor-targeting capacity of the hybrid membranes.

To verify the activity of collagenase, tumors were subjected to Masson’s trichrome staining and immunofluorescence analysis. As shown in Fig. 7c and d, MP@H-MnO_2_-Col NPs and MP@H-MnO_2_-Dox-Col NPs exhibited greater collagen degradation ability than the saline group, indicating that Col was well-retained on the NPs and exhibited strong enzymatic activity. Further, immunofluorescence analysis for Hypoxyprobe-1 was performed to confirm that hypoxia was alleviated in tumors through the MnO_2_-catalyzed generation of O_2_ from H_2_O_2_ (Fig. 7e). Tumors of mice from both the Dox and saline groups exhibited strong green fluorescence, indicating hypoxia. The green fluorescence in the tumors of MP@H-MnO_2_-Dox NPs-treated mice was weaker than that of H-MnO_2_-Dox NPs-treated mice, indicating that MP-coated NPs may undergo greater aggregation to then generate more O_2_ at the tumor site. In particular, the MP@H-MnO_2_-Dox-Col NPs exhibited the weakest green fluorescence, confirming the role of Col in breaking down collagen and promoting greater NPs tumor infiltration. Fig. S1 showed the semi-quantitative analysis of hypoxia positive areas based on 15 confocal images per group. As shown in Fig. S1, MP@H-MnO_2_-Dox-Col NPs group could significantly reduce tumor hypoxia as compared with Dox group (*p* < 0.001).

## Discussion

Hypoxia, acidosis and H_2_O_2_-rich conditions, condensed ECM in TME as well as low targeted ability bring challenges for Dox treatment in breast cancer. It was reported that early 50% of locally advanced breast cancers exhibit hypoxic tissue areas in the tumour mass, and hypoxia is known to directly or indirectly increase Dox resistance on breast cancer cells leading to treatment failure [58]. Moreover, hypoxia induces a metabolic shift causing acidosis and H_2_O_2_-rich conditions in the TME also bring obstacles for Dox therapy. To solve these problems, we developed biomimetic nanosytem for Dox delivery based on MnO_2_ NPs due to their following advantages. (1) MnO_2_ NPs have a high specificity and reactivity toward H_2_O_2,_ generating O_2_in situ and attenuating hypoxia and regulation of pH; (2) MnO_2_ NPs are decomposed to water-soluble Mn^2+^ ions with bio-safety, avoiding the *in vivo* accumulation of the metal oxide commonly observed for other metal-based nanosystems; (3) MnO_2_ NPs have a large specific surface area and high pore volume, providing excellent drug loading and delivery properties. Our results showed that Dox loading in MnO_2_ based nanosystem reached stable values of up to 87%, and MnO_2_-containing NPs producing O_2_, significantly alleviating hypoxia *in vitro* and *in vivo* within the tumor site.

Moreover, the condensed ECM with high collagen content hindering the diffusion of NPs to deeper tumor sites, as well as low targeted ability are the other two obstacles for Dox delivery with loss of Dox therapeutic efficacy and side effects including cardiotoxicity. Here, we used Col to modify NPs, and further covered the NPs via a fusion membrane of inflammation-targeted RAW264.7 cell membrane and pH-sensitive liposomes. The *in vitro* and *in vivo* studies indicated that this Col modified NPs improved the penetration and retention of nanosystem in deep tumor tissue. Additionally, with the help of fusion membrane’s cancer-homing and inflammation targeting ability, MP@H-MnO_2_-Dox-Col obtained tumor targeted ability with enhanced efficacy and low cardiotoxicity of Dox.

In conclusion, we have successfully developed a multifunctional MP@H-MnO2-Dox-Col for breast cancer therapy. Our studies demonstrated that this nanosystem could be targeted delivery to tumor with a good retention ability based on the MP’s cancer-homing and inflammation targeting ability. Furthermore, with the help of Col and H-MnO2, MP@H-MnO2-Dox-Col could alleviate tumor hypoxia and improve the penetration, significantly enhancing the efficacy of Dox for breast cancer without obvious cardiotoxicity. All the encouraging results indicate that MP@H-MnO2-Dox-Col provides a potential new strategy for chemotherapy against breast cancer. Combination of this nanosytem and clinical first-line therapy could be considered in breast cancer therapy in the future.

## Materials and methods

### Materials

Tetraethyl orthosilicate (TEOS), sodium carbonate (Na_2_CO_3_), and doxorubicin hydrochloride (Dox) were obtained from Aladdin Industrial Co. (Shanghai, China). Hydrogen peroxide (H_2_O_2_) (30 wt %) and potassium permanganate (KMnO_4_) were purchased from Sangon (Shanghai, China). [Ru(dpp)_3_]Cl_2_ (RDPP) was obtained from Leyan (Shanghai, China). Lecithin, cholesterol, and DSPE-PEO_Z_ were purchased from Xi’an Ruixi Biological Co., Ltd. (Xi’an, China). (3-aminopropyl)-triethoxysilane (APTES), dimethyl sulfoxide (DMSO), and collagenase type I were purchased from Sigma-Aldrich Co. LLC. (St. Louis, MO, USA). The Collagenase Assay Kit was purchased from Thermo Fisher Scientific (Waltham, Massachusetts, USA). The bicinchoninic acid (BCA) protein assay kit and 4′,6-diamidino-2-phenylindole (DAPI) were obtained from Yeasen (Shanghai, China). 1,1′-dioctadecyl-3,3,3′,3′-tetramethylindotricarbocyanine iodide (DiR) was purchased from Maokang Biotechnology Co., Ltd. (Shanghai, China). 3,3′-dioctadecyloxacarbocyanine perchlorate (DiO) was obtained from Beyotime (Shanghai, China). 1,2-dioleoyl-sn-glycero-3-phosphoethanolamie-N-(lissamine rhodamine B sulfonyl) (DOPE-RhB, 18:1 Liss Rhod, λex/λem = 560/583 nm) and N-[6-[(7-nitro-2-1,3-benzoxadiazol-4-yl) amine] hexanoyl]-phytosphingosine (C6-NBD, λex/λem = 460/534 nm) were purchased from Avanti Polar Lipids (Birmingham, AL, USA). N-hydroxysuccinimide (NHS) and 1-(3-dimethylaminopropyl)-3-ethylcarbodiimide hydrochloride (EDC) were purchased from Aladdin. The CytoTox 96® Non-Radioactive Cytotoxicity Assay Kit was purchased from Promega (Madison, Wisconsin, USA).

RAW264.7 macrophage cells were purchased from the Institute of Biochemistry and Cell Biology (Shanghai, China). Phosphate-buffered saline (PBS), RPMI 1640 medium, Dulbecco’s modified Eagle’s medium (DMEM), and fetal bovine serum (FBS) were purchased from Gibco (New York, USA). All other chemicals were of analytical grade and were used without further purification.

Female BALB/c mice (6–8 weeks old) were purchased from Jessie Experimental Animal Co., Ltd. (Shanghai, China).

### Synthesis and characterization of MP@H-MnO_2_-dox-col NPs

H-MnO_2_ NPs were synthesized following a template etching method. The transition from solid silica to mesoporous manganese dioxide was observed using TEM Images.

### Synthesis of H-MnO_2_ NPs

The H-MnO_2_ NPs were synthesized as per a previously reported method [59]. Firstly, the template solid silica nanoparticles (sSiO_2_ NPs) were synthesized. To this end, 14 mL of ethanol, 2 mL of deionized water, 500 µL of NH_3_·H_2_O (28%), and 100 µL of TEOS were mixed in a 50 mL conical flask and stirred for 2 h at 45 °C. sSiO_2_ NPs were collected by centrifugation at 11,000 rpm and washed with ethanol and water thrice. sSiO_2_@MnO_2_ NPs were synthesized as follows: KMnO_4_ solution (600 mg, 20 mL) was added dropwise into the prepared sSiO_2_ NPs solution in a water bath sonicato (KQ-500E ultrasonic cleaner, Kunshan Ultrasonic Instruments Co. Ltd., China), stirring for 12 h after dropping, and the products were collected via centrifugation at 11,000 rpm. Finally, sSiO_2_@MnO_2_ NPs were etched with Na_2_CO_3_ (2 M) solution at 60 °C for 12 h to obtain H-MnO_2_ NPs.

### Preparation of H-MnO_2_-dox-col NPs

For Dox loading, the H-MnO_2_ solution (0.2 mg·mL^− 1^) was mixed with different concentrations of Dox for 12 h. The obtained H-MnO_2_was functionalized with amino groups by using APTES. Dox (6 mg) was mixed with H-MnO_2_ solution (0.2 mg·mL^− 1^) and stirred for 12 h to form H-MnO_2_-Dox. The carboxyl group in Col (4 mg) was activated using EDC (95 mg) and NHS (57 mg) for 30 min and then stirred with H-MnO_2_-Dox NPs for 24 h at 4 °C. The H-MnO_2_-Dox-Col NPs were obtained after centrifugation and washing.

### Preparation of hybrid pH-sensitive fusion membrane

The RAW264.7 cell plasma membranes were separated following a previously reported method of repeated freezing and thawing [60]. Briefly, the membrane protein extraction kit (Beyotime Biotechnology, Shanghai, China) was used to extract M. The RAW264.7 cells was collected with a scraper, washed, centrifuged at 300 × g for 5 min, and further counted. Cells were suspended in membrane protein extraction reagent A and kept in an ice bath for 15 min as per kit instructions. The cell-containing solution was then repeatedly freeze-thawed three times in liquid nitrogen at 25℃. The obtained solution was centrifuged at 4℃ and 700 × g for 10 min, and the supernatant was further centrifuged at 4℃ and 14,000 × g for 30 min to obtain the precipitate, which was the cell membrane fragments. The BCA kit was used to analyze the protein content of membranes for the subsequent preparation of MP@H-MnO_2_-Dox-Col NPs, MP@H-MnO_2_-Dox NPs, and MP@H-MnO_2_-Col NPs.

To fabricate MP, P was first prepared via thin-film hydration as previously reported [61]. Briefly, 3.1 mg of lecithin, 0.8 mg of cholesterol, and 2.2 mg of DSPE-PEOz were dissolved in dichloromethane (6 mL) in a round-bottom flask, and the mixture was evaporated to form a transparent film. The film was hydrated with 2.8 mL of PBS and 200 µL of M suspension or with 3 mL of PBS to prepare MP or P. The suspension was sonicated and extruded through 0.2 and 0.1 μm polycarbonate membranes for further optimization.

### Fusion study of M and P

M and P fusion was observed using Förster resonance energy transfer (FRET)[40, 62]. Briefly, M was labeled with DOPE-RhB and C6-NBD, and P was fused with labeled M at different mass ratios (0:1, 1:1, 2:1, 3:1, 4:1, and 5:1). A 500–600 nm spectrum excited by 470 nm was recorded to monitor fluorescence recovery based on the donor (C6-NBD). Additionally, Fourier transform infrared (FT-IR) spectroscopy analysis was used to determine the membrane component M, P and MP and to verify MP fusion.

### Optimized membrane coating study

In order to explore the optimal ratio of fusion membrane and H-MnO_2_ NPs, we incubated MP with H-MnO_2_ NPs at different weight ratios (w/w) from 1:5 to 5:1 and then sonicated the mixed NPs for 2 min. Subsequently, the uncoated hybrid membrane fragments were removed via centrifugation for 30 min at 11,000 rpm and 4 °C. To assess the optimized weight ratio, the surface membrane protein content of MP@H-MnO_2_ NPs was determined using the BCA kit.

### Characterization of MP@H-MnO_2_-dox-col NPs

The MP was coated onto the core H-MnO_2_-Dox-Col via sonication for 5 min to form MP@H-MnO_2_-Dox-Col NPs. The hydrodynamic diameter and zeta potential of NPs at different stages of synthesis were determined by dynamic light scattering (DLS) (ZetaSizer Nano ZS90, Malvern Instruments, UK) at 25 °C. In addition, the morphology of the MP@H-MnO_2_-Dox-Col NPs was observed using transmission electron microscopy (TEM) (TECNAI G2 S-TWIN, FEI, USA). MP@H-MnO_2_-Dox-Col NPs (2 mg) were dispersed in 2 mL of PBS at pH = 6.5 for 2 h, prior to observation via TEM in order to study the pH-triggered properties of MP@H-MnO_2_-Dox-Col NPs. Finally, H-MnO_2_-Dox NPs and MP@H-MnO_2_-Dox-Col NPs were re-suspended in PBS, and the particle sizes over time were measured via DLS within 15 days in order to investigate the stability of NPs. Various NP formulations were analyzed using UV/VIS/NIR (Shimadzu, Kyoto, Japan) spectroscopy to further verify the successful synthesis of MP@H-MnO_2_-Dox-Col NPs.

### Protein characterization

The protein profiles of M, MP, and MP@H-MnO_2_-Dox-Col NP were evaluated via sodium dodecyl sulfate-polyacrylamide gel electrophoresis (SDS-PAGE) and coomassie brilliant blue staining (Beyotime, Shanghai, China). Western blotting was conducted to identify specific protein markers in RAW264.7 cells. After transferring the proteins onto polyvinylidene difluoride membranes, these were incubated at 4℃ overnight with anti-histone H3 (BioLegend, San Diego, CA, USA), anti-pan-cadherin (Santa Cruz Biotechnology, Dallas, TX, USA), and integrin α4 antibodies (Proteintech, Chicago, USA) and Na^+^-K^+^-ATPase (Abcam, Cambridge, UK), which was used as a reference protein.

### Confocal microscopy of pH-sensitive hybrid fusion membranes

Confocal microscopy was used to observe membrane colocalization. Briefly, M was stained with DiR (excitation/emission: 748/780 nm), and P was dyed with DiO (excitation/emission: 484/501 nm). These labeled membranes were used to prepare MP@H-MnO_2_-Col NPs, M@H-MnO_2_-Col NPs, and P@H-MnO_2_-Col NPs. Their colocalization was evaluated under a confocal laser-scanning microscope (CLSM) (Olympus, Tokyo, Japan).

### ***In vitro*** pH and H_2_O_2_-stimulated drug release

To observe the release behavior of Dox from MP@H-MnO_2_-Dox-Col NPs, 2 mL of MP@H-MnO_2_-Dox-Col NPs solution was added into a dialysis bag (MWCO = 3500 D) with or without 100 µM H_2_O_2_ at pH = 7.4 or pH = 6.5 and then immersed in 50 mL of PBS, with stirring at 100 rpm and 37.0 °C. At the specified time points, 3 mL of solution was removed to measure the absorbance via UV-vis spectrometry (APL, Shanghai, China) and replaced with an equal volume of release medium.

### ***In vitro*** detection of O_2_ generation

The generated O_2_ was detected using an RDPP probe, whose fluorescence could be strongly quenched by O_2_ [63]. Briefly, 1 mL Dox + Col, H-MnO_2_-Dox, and MP@H-MnO_2_-Dox-Col NPs (50 µg mL^− 1^) were uniformly suspended in pH 6.5 PBS solution. Then, 50 µL RDPP ethanol solution (0.01 M) was added and stirred for 5 min, whereafter 250 µL H_2_O_2_ (100 mM) was added. The fluorescence intensity of RDPP was recorded at an emission wavelength of 615 nm at the designated time points.

### Determination of collagenase enzymatic activity

The enzyme activities of several NP formulations were tested as per a previously described method [32, 33]. For this experiment, 60 µL of collagenase assay buffer, 40 µL of collagenase substrate, and 100 µL of sample reaction were added to 96-well plates. A microplate reader (Thermo Fisher Scientific, Waltham, MA, USA) was used to measure the absorbance at 345 nm.

### Biocompatibility evaluation

The cytotoxicity of MP@H-MnO_2_-Col NPs was evaluated using a CCK-8 kit. 4T1 cells were seeded in 96-well plates (8,000 cells/well), and incubated with the vector at 0–167 µg·mL^− 1^ for 24 h. Absorbance was measured at 450 nm using a microplate reader (Thermo Fisher Scientific, USA).

### Intracellular O_2_ evaluation

O_2_ generated by MP@H-MnO_2_-Dox-Col NPs was measured using a [Ru(dpp)_3_]Cl_2_ probe. 4T1 cells (10^5^ cells/dish) were cultured in confocal dishes in a normoxic or hypoxic atmosphere for 24 h. To evaluate the alleviation of intracellular hypoxia, MP@H-MnO_2_-Dox-Col NPs (MnO_2_: 20 µg·mL^− 1^) were added to the cells in a hypoxic environment for 6 h, followed by incubation with the [Ru(dpp)_3_]Cl_2_ probe for another 6 h. The cells were washed with PBS and observed using a CLSM.

### ***In vitro*** cellular uptake measurement

A CLSM was used to evaluate the distribution of NPs in 4T1 cells. 4T1 cells (10^5^ cells/well) were seeded onto coverslip-covered 24-well plates and incubated overnight, followed by treatment with free Dox, H-MnO_2_-Dox NPs, MP@H-MnO_2_-Dox NPs (pH = 7.4 or 6.5), or MP@H-MnO_2_-Dox-Col NPs (pH = 7.4 or 6.5) at an equal dose of 1 µg/mL Dox for 4 h. Next, 4T1 cells were washed with PBS and fixed with 4% paraformaldehyde, while DAPI was used for nuclear staining. The cells were then observed using a CLSM.

### Tumor spheroid culture

Three-dimensional (3D) tumor spheroids were developed as per the liquid-covering method, with slight modifications [64]. Briefly, 96-well plates were coated with sterile 50 µL of 1% agarose. Approximately, 2 × 10^3^ 4T1 cells were then seeded in each well and cultured for approximately 5 days at 37 °C with 5% CO_2_. The growth of the spheroids was observed under a microscope. When the diameter of the spheroids reached approximately 250 μm, subsequent spheroid experiments were performed.

### ***In vitro*** assessment of the antitumor effect

To assess the growth of tumor spheroids, the following solutions with Dox concentration of 5 µg/mL were added to a 24-well plate : PBS, MP@H-MnO_2_-Col NPs, Dox, H-MnO_2_-Dox NPs, MP@H-MnO_2_-Dox NPs (pH = 7.4 or 6.5), and MP@H-MnO_2_-Dox-Col NPs (pH = 7.4 or 6.5). After 1, 3, 5, or 7 days of incubation, the size of the tumor spheroids was assessed using a fluorescence microscope (Zeiss LSM 510, Thornwood, NY, USA).

To evaluate the cytotoxicity of these solutions, the lactate dehydrogenase (LDH) assay was performed. Dox, H-MnO_2_-Dox NPs, MP@H-MnO_2_-Dox NPs (pH = 7.4 or 6.5), and MP@H-MnO_2_-Dox-Col NPs (pH = 7.4 or 6.5) (5 µg·mL^− 1^) were added to 5-day-old spheroids growing in 96-well plates and incubated for 72 h. Cytotoxicity was measured using the CytoTox 96® Non-Radioactive Cytotoxicity Assay Kit.

### Assessment of ***in vitro*** NP penetration in 3D tumor spheroids

Five-day-old spheroids were transferred to confocal dishes and treated with various NPs at pH 7.4 or pH 6.5 for 4 h. Z-stack scanning was performed on the 3D tumor spheroids from top to bottom with 5 μm per section using a CLSM. Several tumor spheroids were imaged synchronously.

### ***In vivo*** tissue distribution of MP@H-MnO_2_-Dox-Col NPs

An in situ breast cancer model was established in female BABL/c mice by injecting 10^6^ 4T1 cells into the right sides of the mammary gland fat pads. The tumors grew to 80–100 mm^3^ on day 7, and the DiR dye was used to investigate the *in vivo* distribution of NPs. For this purpose, the mice were intravenously injected with free DiR, H-MnO_2_-DiR, or MP@H-MnO_2_-DiR-Col (equivalent to 5 mg·kg^− 1^ DiR, n = 5 for all groups) through the tail vein and observed at 1, 4, 12, and 24 h post-injection using the IVIS imaging system (Xenogen IVIS-200, Caliper Life Sciences, Hopkinton, MA, USA) at 748/780 nm. The mice were sacrificed at 24 h post-injection. Major organs (the heart, liver, spleen, lungs, and kidneys) and tumors were isolated and collected for *ex vivo* fluorescent imaging. All the data were analyzed using Quick View 3000 software.

### ***In vivo*** antitumor effect study

One week after establishing the in situ breast cancer model as previously described, the mice were randomly divided into six groups (n = 5) and received the following formulations through the tail vein every 3 days for a total of five times: (i) saline (control); (ii) MP@H-MnO_2_-Col NPs; (iii) Dox; (iv) H-MnO_2_-Dox NPs; (v) MP@H-MnO_2_-Dox NPs; (vi) MP@H-MnO_2_-Dox-Col NPs (equivalent dose of Dox, 5 mg·kg^− 1^). The first dose was recorded as the first day. Tumor volume and body weight were measured every 2 days. The following formula was used to calculate the tumor volume: V (mm^3^) = a × b^2^/2, in which a and b are the minimum and maximum diameter, respectively. Additionally, the above treatment drugs were injected through the caudal vein to the breast cancer model mice, and the survival of mice was recorded.

After the experiment, all animals were sacrificed. Tumor tissues and major organs (the heart, liver, spleen, lungs, and kidneys) were carefully excised, weighed, and subjected to hematoxylin and eosin (HE), Ki-67, and terminal deoxynucleotidyl transferase dUTP nick end labeling (TUNEL) staining.

### ***In vivo*** collagen fiber digestion and NPs penetration

Immunofluorescence staining and Masson’s trichrome staining were used to evaluate the degradation of collagen by NPs in tumor tissues. After mice were treated with different NP formulations as mentioned above, the tumors were separated, fixed, and embedded. The sections were then incubated with an anti-collagen I antibody, anti-CD31 antibody, and DAPI, respectively, followed by observation using a slice scanner. In addition, Masson’s trichrome staining was performed using a commercial kit. The imaging of tumor tissue was performed using a microscope (Aperio VERSA, Leica, USA). The penetration of NPs *in vivo* was analyzed by observing the distribution of Dox autofluorescence when scanning the tumor sections.

### Investigation of hypoxia ***in vivo***

To evaluate hypoxia alleviation *in vivo*, Hypoxyprobe™-1, a substituted 2-nitroimidazole with the chemical name of pimonidazole hydrochloride, was used. Pimonidazole hydrochloride (60 mg·kg^− 1^) was injected intraperitoneally, and the tumors were dissected 90 min later. The cell nuclei and hypoxic areas of tumor sections were stained with DAPI and an anti-pimonidazole antibody, followed by observation under a microscope.

### Statistical analysis

Prism software (version 7.0) was used to analyze the experimental results. Data are presented as the mean ± SD. The significance of differences between groups was analyzed using ANOVA. Statistical significance is shown as *p < 0.05, **p < 0.01, and ***p < 0.001.

## Electronic supplementary material

Below is the link to the electronic supplementary material.

Supplementary Material 1

## Data Availability

All data generated or analyzed during this study are included in this published article.
