# Supplementary material for: Biomimetic liposomal nanozymes improve breast cancer chemotherapy with enhanced penetration and alleviated hypoxia
Source: J Nanobiotechnology. 2023 Apr 10;21:123. doi: 10.1186/s12951-023-01874-7 (PMC10084658; doi:10.1186/s12951-023-01874-7)
Supplement: Supplementary file 1 — Supplementary Material 1 [file 12951_2023_1874_MOESM1_ESM.doc]

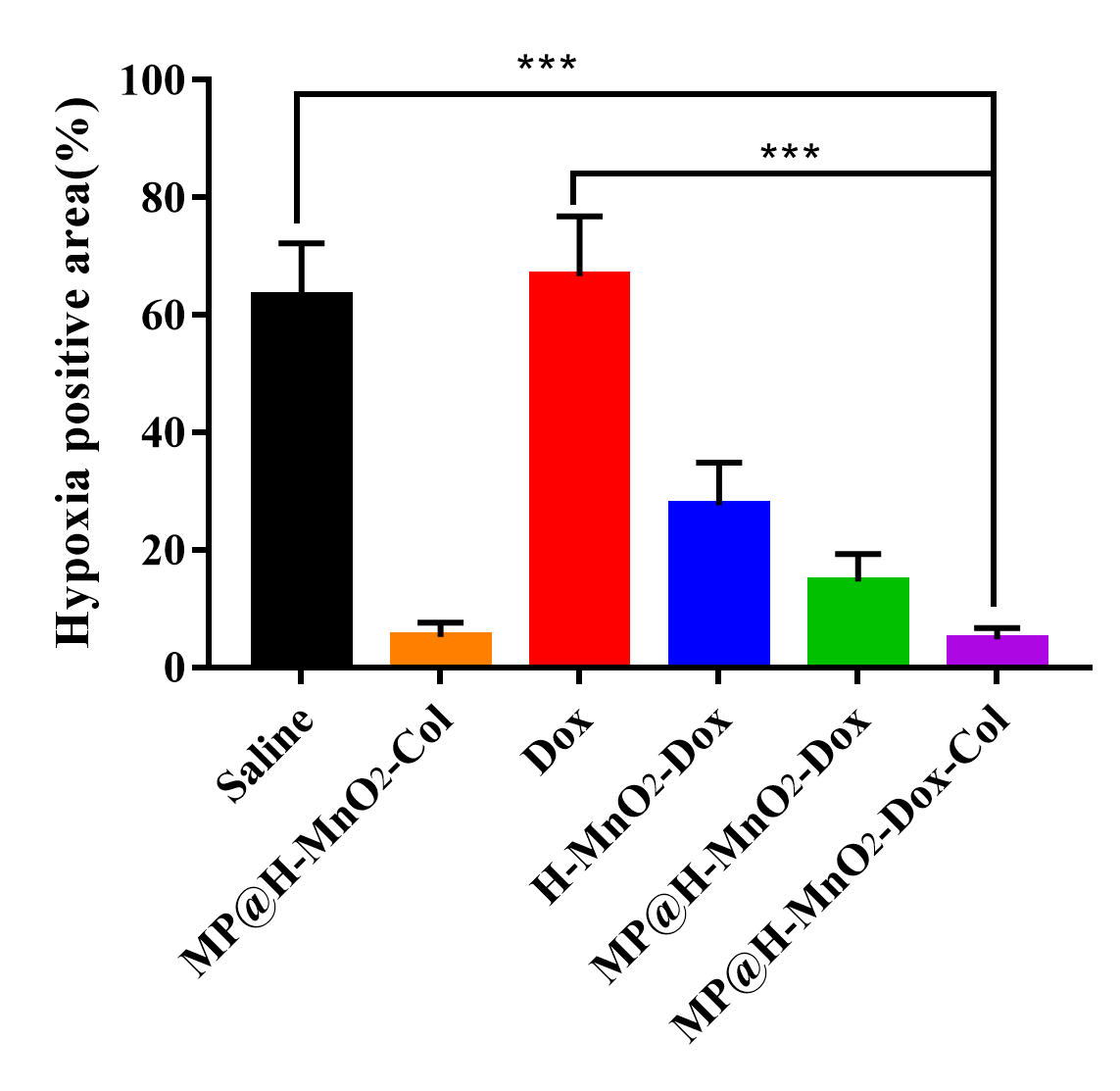


**Fig. S1** Quantitative analysis of tumor hypoxic areas post injection of various nanoparticles. Data are presented as the mean ± SD (n = 15). ****p* < 0.001
